# The oncogenic miR-429 promotes triple-negative breast cancer progression by degrading DLC1

**DOI:** 10.18632/aging.205051

**Published:** 2023-09-21

**Authors:** Yao Li, Xue Meng, Yuqing Luo, Shuai Luo, Jin Li, Jiafei Zeng, Xiang Huang, Jinjing Wang

**Affiliations:** 1 Department of Pathology, Affiliated Hospital of Zunyi Medical University, Zunyi, Guizhou Province, P.R. China

**Keywords:** triple-negative breast cancer, miRNAs, miR-429, DLC1

## Abstract

Lines of evidence have demonstrated that the oncogenic miRNAs are pivotal to the progression of breast cancer. In this study, we investigated the biological traits of microRNA-429 (miR-429) in triple-negative breast cancer (TNBC) and the underlying molecular mechanism. We found that miR-429 was notably overexpressed in TNBC, and promoted TNBC cell proliferation, migration, and invasion by degrading the tumor suppressor DLC1. In conclusion, our findings reveal the mechanism of tumorigenic miR-429 in TNBC, which paves the way for target therapies translation in clinical settings.

## INTRODUCTION

Breast cancer is a highly predominant malignancy in females globally and poses a significant threat to their health [1]. Regarded as a subtype of breast malignancy, TNBC is featured with a lack of human epidermal growth factor receptor 2 (HER-2), progesterone receptor (PR), and estrogen receptor (ER) expression, accounting for almost 15%-20% of breast cancers [2]. TNBC is frequently diagnosed in young patients and is associated with high histological grade and invasive histology. It has a poor prognosis, high recurrence metastasis rate and mortality [3–6], and lacks effective endocrine and targeted therapies.

Hepatocellular carcinoma deletion gene 1 (DLC1) is an oncogene located on human chromosome 8p22. Its downregulated expression is commonly shown in breast cancer [7]. Meanwhile, Zhou et al. suggested that DLC1 is also associated with genes related to apoptosis, cell cycle progression and proliferation in BC cells. Those indicate DLC1 is one of the most essential tumor suppressors on 8p22 [7].

Constituting around 19-24 nucleotides, the endogenous non-coding small RNAs known as microRNAs (miRNAs) are significant in their performance as cis-acting elements of gene expression. They act in a regulatory capacity mainly by degrading or inhibiting the translation of target mRNAs [8]. Many studies have shown that miRNA dysfunction interferes with the expression of the tumor suppressor target gene and participates in the modulation of cell proliferation, apoptosis, invasion, and migration, playing a crucial role in tumorigenesis and development [9, 10]. Fang et al. [11] employed quantitative real-time polymerase chain reaction (qRT-PCR) to demonstrate the considerable upregulation of miR-21 in TNBC cell lines and tissues. Subsequently, they observed that the downregulation of miR-21 reduced the invasive ability of TNBC cells and their proliferation. Dual-Luciferase assays were also conducted to confirm the direct binding of miR-21 to PTEN 3’UTR, which resulted in the negative modulation of Phosphatase and tensin homolog (PTEN) protein expression. These data provide valuable insights into potential therapeutic targets and prognostic indicators for TNBC patients.

The miR-429 is implicated in various aspects of tumorigenesis and progression [12]. Dai et al. [13] analyzed the miRNA expression data in breast cancer samples in the database The Cancer Genome Atlas (TCGA). The data indicated increased expression of miR-429 in breast cancer tissues, which varied across different breast cancer cell lines in humans. Additionally, the upregulation of miR-429 *in vitro* promoted the growth of malignant breast cells and highlighted its potential as a diagnostic and prognostic marker for breast cancer; however, this study did not investigate the underlying mechanism. At present, the biological effects of miR-429 on TNBC and its mechanism of action are still unclear Therefore, it is necessary to further explore the function of miR-429 in the development of TNBC and the regulatory role of its target genes to provide new strategies for the treatment of TNBC.

## MATERIALS AND METHODS

### Bioinformatical analysis

The database TCGA (https://portal.gdc.cancer.gov/) was utilized to assess the expression profiles of mRNA (normal: n= 13, tumor: n = 175) and mature miRNA (normal: *n =* 102, tumor: *n* =63) of TNBC, by using “edgeR” package [14]. The DESeq2 was utilized to execute differential analysis and the differentially expressed mRNAs (DEmRNAs) were screened per a specific criterion (|Log2FC|>1, *p*adj<0.01). The TargetScan (http://www.targetscan.org/vert_72/), miRTarBase (https://mirtarbase.cuhk.edu.cn/~miRTarBase/miRTarBase_2022/php/index.php), and miRDB (http://mirdb.org/) databases were utilized for the identification of the potential targets which were further screened out based on their differential downregulation in the TCGA. Additionally, Pearson correlation analysis was conducted to determine notable correlation coefficients between miR-429 and mRNA targets.

### Cell culture and transfection

Healthy human mammary epithelial cells MCF-10A (no. CTCC-001-0286) were cultured with MEBM BulletKit (Gibco, Lonza, Basel, Switzerland), whereas human TNBC cell line MDA-MB-468 (no. CTCC-001-0009) and 293T cells were cultured in DMEM Medium-H (Gibco, Carlsbad, CA, USA) supplemented with 10% fetal bovine serum (FBS; Gibco™, USA),100 μg/ml streptomycin, and 100 units/ml penicillin (HyClone™, Logan, UT, USA). The cells were then kept under 5% CO_2_ in an incubator with a temperature of 37° C. All cell lines were purchased from Meisen CTCC (Zhejiang, China) and authenticated by STR.

The design and synthesis of miR-429 mimic, miR-429 inhibitor, and its negative control (NC) were executed by GENE (Shanghai, China). Furthermore, DLC1 overexpression plasmid (pcDNA-*DLC1*) was constructed using pcDNA3.4 vector and DLC1 interference RNA (si*DLC1*) was synthesized by the OBiO company (Shanghai, China). Following the inoculation of MDA-MB-468 cells into 6-well plates for 12 h. Then, two 1.5ml sterilized EP tubes were taken and 100μl serum-free culture solution was added to each tube. The specified transfected 2.5ug plasmid or 100nm RNA was added to one EP tube, and 5μl Lipofectamin2000 transfection reagent (Invitrogen, Carlsbad, CA, USA) was added to the other EP tube. After being placed for 5 minutes, the two EP tubes were mixed and shaken well, and then left for 20 minutes. Finally, the above mixture was added to the six-well plate 0.2ml/ well for further culture. These cells were afterwards collected for expression analysis and functional experiments.

### qRT-PCR

The extracted total RNA from the selected cells was converted into cDNA using RNAiso Plus reagent (Takara, Dalian, China) and PrimeScript Reverse Transcription Detection Kit (Takara, Dalian, China), respectively. Amplification of the cDNA was executed through the SYBR Green Master Mix kit (Takara, Dalian, China). The miR-429 and *DLC1* mRNA expression levels were assessed using U6 and GAPDH as internal references. The relative expression was expressed as 2^-ΔΔCt^ values and the primer sequences are enlisted in Table 1.

**Table 1:** Primer sequences for qRT-PCR.

**Gene**	**Primer sequence (5′-3′)**
miR-429	Forward: CGCGCGTAATACTGTCTGGTAA
	Reverse: AGTGCAGGGTCCGAGGTATT
U6	Forward: GCTCGCTTCGGCAGCACATATAC
	Reverse: AGTGCAGGGTCCGAGGTATT
DLC1	Forward: CGAGATCTTCCTGAGCCACTAAT
	Reverse: GCTGTGACATCGCTCAGGAAATA
GAPDH	Forward: ACAACTTTGGTATCGTGGAAGG
	Reverse: GCCATCACGCCACAGTTTC

### Western blot

RIPA lysis buffer (PMSF; R0010; Solarbio Science and Technology, Beijing, China) with the addition of phenylmethylsulfonyl fluoride was utilized for the extraction of the cellular proteins. The specific concentrations of the proteins were quantified by a BCA protein assay kit (Sangon Biotech, Shanghai, China). Furthermore, separation of the protein samples was executed by SDS-PAGE (Sangon Biotech, Shanghai, China), which were shifted onto PVDF membranes (0.45μm, Merck, GER). To block the membranes, they were treated at room temperature with 5% skimmed milk for 1h. Overnight incubation of the rabbit polyclonal antibody against DLC1 (ab126257, 1:1000, Abcam, UK) and GAPDH (1:5000, Proteintech, China) with the membrane was executed at 4° C. These were subsequently rinsed thrice with TBST (Tris-buffered saline with Tween 20) for about 15 min each. Followed by room temperature incubation with anti-rabbit IgG (HR P-linked; 1:2000, Sangon Biotech, China) for 1 h. These were then cleansed thrice with TBST (15 min each). Finally, ECL chromogenic solution was applied to the membrane and the image was developed by gel imager. The resulting data were analyzed by Image Lab software.

### CCK-8 assay

The transfected MDA-MB-468 cells (2×10^3^ cells/well) were inoculated into 96-well plates. The proliferative capacity of the cells was detected by the CCK-8 assay kit (Dojindo Laboratory, Tokyo, Japan) at 0, 24, 48, and 72 h. Following the addition of CCK-8 solution (10 μl per well), the absorbance of the cells was assessed at 450 nm with an enzyme marker after incubation at 37° C for 90 min.

### Wound healing assay

The migratory capacity of the cells was examined through the wound healing assay. MDA-MB-468 cells (4×10^5^ cells/well) were inoculated into 6-well plates and transfected for 24 h. To initiate the assay, the cells were scratched in a vertical direction using a 100 μl pipette, with subsequent rinsing of the cells with phosphate-buffered saline (PBS) (twice). These were then shifted to a serum-free medium, with photographs taken under a microscope to record the initial wound distance (0 h). The plates were further incubated for 24 h. Afterwards, the 6-well plates were removed and photographed under a microscope and the 24 h scratch distance was recorded. The relative migration rate of cells was calculated adapted from the formula referred to Bahar et al. [15], i.e., would closure (%) = W_0_ – W_t_/W_0_ × 100, Healing speed (*μ*m^2^/h): W_0_ – W_t_ /ΔT; Relative wound area: W_0_ /W_t_. W_0_: wound area at 0 h (μm^2^); W_t_: wound area at ∆h (μm^2^); ∆T: duration of wound measured (h).

### Transwell assay

After 24 h of transfection, MDA-MB-468 cells were seeded in the upper portion of the transwell chamber containing matrix gel (pore size 8 μm, 3422, Corning, NY, USA) with 200 μL of serum-free cell suspension (8×10^4^ cells) added. The addition of 500 μL of DMEM complete medium with 10% fetal bovine serum to the lower chamber was followed by 24 h incubation of the cells. Afterwards, gentle removal of the cells remaining on the surface of the upper chamber was carried out with a cotton swab. Subsequently, 4% paraformaldehyde was utilized for fixation of the infiltrated cells in the lower chamber for 30 min, which were then stained with 0.1% crystal violet. The number of cells invaded was counted through a microscope.

### Dual-luciferase reporter assay

cDNA fragments of the DLC1 3′UTR containing the miR-429 binding site were amplified by PCR and inserted into the psiCHECK(TM)-2 luciferase vector (Wuhang, China), contributing to the construction of the wild type (WT) DLC1 (DLC1 3′UTR-WT). The mutant (MUT) DLC1 (DLC1 3′UTR-MUT) was constructed using the fast site-directed mutagenesis kit (Agilent, Roseville City, CA, USA). Respectively, co-transfection of 293T cells with WT or MUT vectors and miR-429 or NC mimics was carried out. The luciferase activity was examined through a dual luciferase reporter assay kit (Promega, Madison, WI, USA) after 48 h.

### Immunohistochemistry (IHC) and hematoxylin and eosin (HE) staining

8 adjacent normal mammary tissues (> 5cm from cancer tissue) and114 cases of formalin-fixed paraffin-embedded (FFPE) tissue blocks of –treatment-naive female patients with TNBC were retrieved from the Department of Pathology, the Affiliated Hospital of Zunyi Medical University from January 2021 to August 2014.TNBC tissues were stained for IHC using a streptavidin-peroxidase-based method or for purple-blue staining of the nucleus with hematoxylin and pink staining of the extracellular matrix and cytoplasm with eosin. Cytoplasmic staining of DLC1 (1:100; ab126257; Abcam, USA) was demonstrated positive; The mean optical density of DLC1 was analyzed by image-Pro Plus 6.0 Image analysis software (Mean optical density (MOD) = integrated optical density /Area). Meanwhile, the overall survival (OS) rate of these TNBC patients was calculated based on in-patient records and follow-ups.

### Statistical methodologies

Statistical analyses were performed by SPSS 27.0 software (IBM Corp., USA). Variables were shown as mean ± standard deviation (SD) unless stated otherwise. Differences between the two groups were conducted by Student’s t-test unless stated otherwise. Two-way repeated measures ANOVA was employed to compare different groups transfected, which measured more than once, including CCK-8 assay. The Kruskal-Wallis test was used to examine the differences in immunostaining scores among the clinical settings, and a false discovery rate (FDR) was utilized to correct the multiple comparisons. Kaplan-Meier curves were utilized to compare the OS rate between “lower” and “high” DLC1 expression based on the median score, and the *p*-value was calculated by log-rank test, by referring to Chen et al. [16]. *P* values were shown as: * p < 0.05, ** p < 0.01, and *** p < 0.001.

## RESULTS

### The tumorigenic feature of miR-429 in TNBC

**Figure 1:**
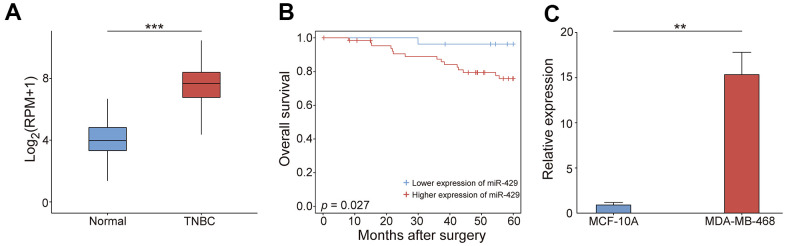
**The tumorigenic feature of miR-429 in TNBC.** (**A**) the miR-429 relative expression in normal (n=102) and TNBC tissues (n=63). (**B**) Kaplan-Meier curve analysis of OS in patients with TNBC (Blue: reduced expression; Red: enhanced expression). (**C**) miR-429 relative expression in MCF-10A and MDA-MB-468 cells. Scale bar = 50 μm. TNBC: triple-negative breast cancer; OS: overall survival. (* p < 0.05, ** p < 0.01, and *** p < 0.001).

This study investigates the process by which miR-429 affects the progression of TNBC. miR-429 expression was assessed using TCGA data and the resulting data indicated that its expression was markedly enhanced in TNBC tissues in contrast to normal tissues (Figure 1A). Survival analysis indicated that individuals with increased miR-429 expression had a considerably shorter 5-year OS than those with reduced miR-429 expression (Figure 1B), therefore, implying the function of miR-429 as an adverse factor in breast cancer prognosis. Next, the expressed miR-429 in TNBC cells was examined and the data indicated a notable increase in miR-429 expression in the TNBC cell line MDA-MB-468 than in normal breast cells MCF-10A (Figure 1C). Overall, these data confirm that miR-429 is considerably elevated in cell lines and tissues of TNBC and is linked with poorer prognoses in TNBC patients.

### miR-429 promotes TNBC cell proliferation, migration, and invasion

**Figure 2:**
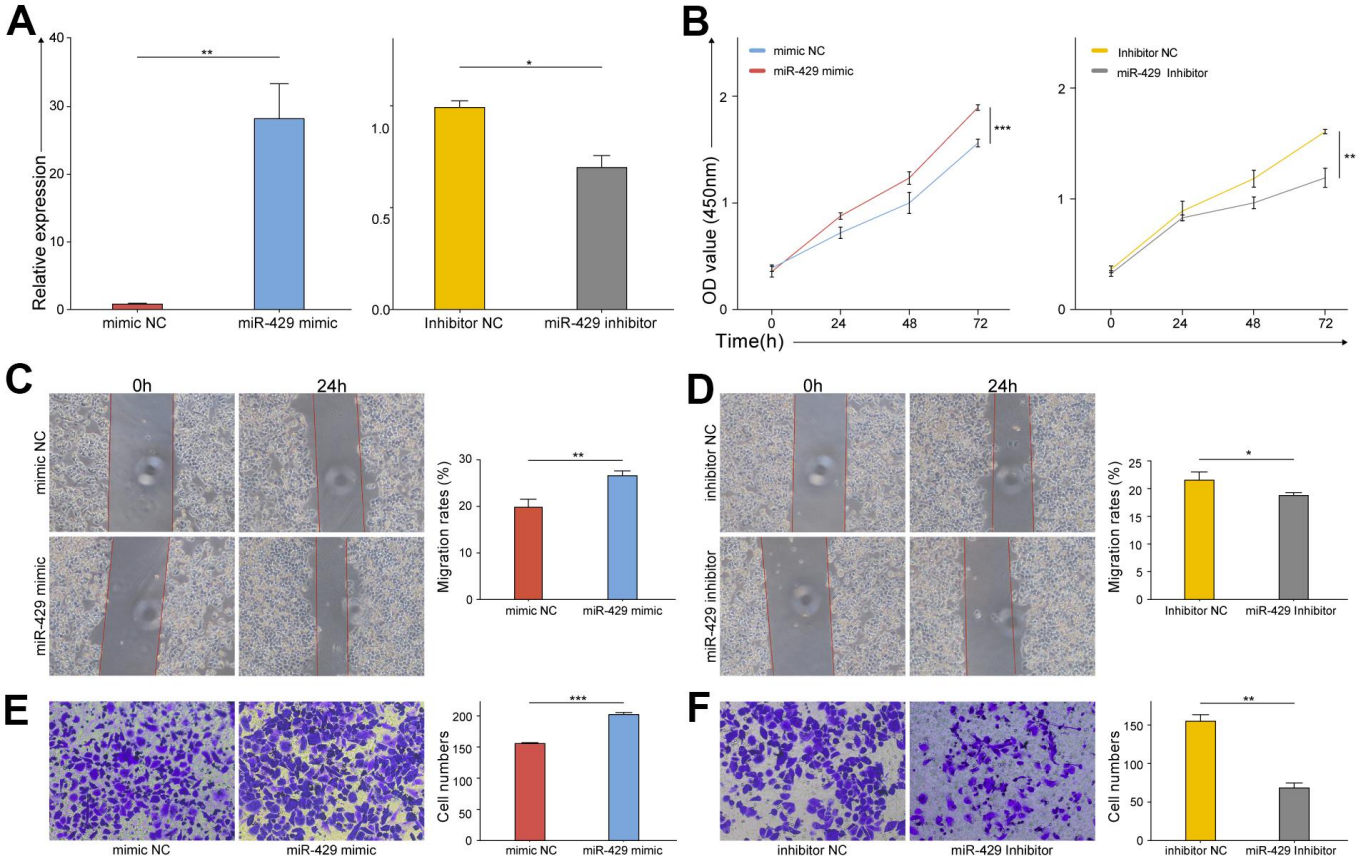
**The proliferation, migration, and invasion of TNBC cell were promoted by miR-429.** (**A**) qRT-PCR analysis of miR-429 expression in TNBC cell transfected with miR-429 mimic, mimic NC, miR-429 Inhibitor or Inhibitor NC; The cell growth curves of (**B**) mimic NC, miR-429 mimic, miR-429 Inhibitor and Inhibitor NC groups were evaluated by CCK8 assays; Wound healing assays in (**C**) mimic NC and miR-429 mimic groups and (**D**) Inhibitor NC and miR-429 Inhibitor groups were executed to detect the cell migration of transfection groups (magnification, × 100). Transwell assays in (**E**) mimic NC and miR-429 mimic groups and (**F**) Inhibitor NC and miR-429 Inhibitor groups were executed to detect the cell invasion of transfection groups (magnification, × 200). All figures are at a scale of 50μm. (* p < 0.05, ** p < 0.01, and *** p < 0.001).

The biological functions of miR-429 were examined through gain- and loss-of-function studies *in vitro*. The knock-down and up-regulation efficiency was evaluated by qRT-PCR, respectively. The study confirms that miR-429 expression was amplified in miR-429 mimic transfected cells and diminished in the miR-429 inhibitor transfected cells (Figure 2A). Various *in vitro* experiments were carried out as well. CCK-8 assays demonstrated an increase in the viability of MDA-MB-468 cells linked to the upregulation of miR-429, while its downregulation decreased cell viability (Figure 2B). Wound healing assay indicated that miR-429 overexpression resulted in a considerably higher percentage of MDA-MB-468 cell healing (Figure 2C), whereas this percentage was considerably lower in the downregulated miR-429 group in contrast to the control (Figure 2D). Transwell assay revealed that elevated expression of miR-429 considerably enhanced the invasive ability of MDA-MB-468 cells (Figure 2E), however, downregulation of miR-429, played the opposite role (Figure 2F). In summary, the upregulated miR-429 notably promoted the growth, migration, and invasion of TNBC cell cells, whereas the downregulation of miR-429 played the opposite role.

### miR-429 targets and negatively modulates DLC1 expression

**Figure 3:**
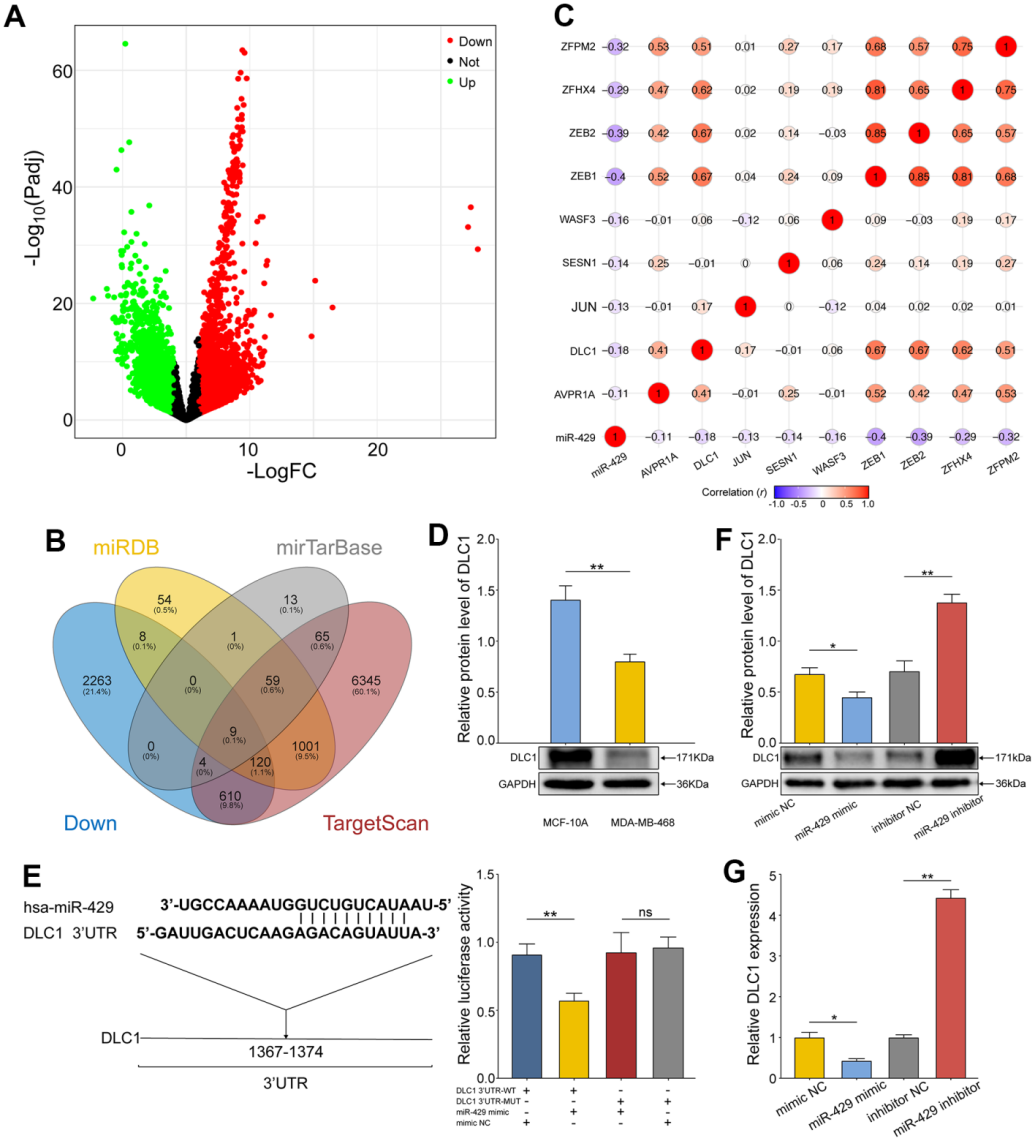
**miR-429 targets and negatively regulates the expression of DLC1.** (**A**) Volcano map of DEmRNAs in TCGA-TNBC data. The upregulated genes with differential expression were in Blue and the downregulated ones were in Red; (**B**) Venn diagram of potential target genes and downregulated DEmRNAs for miR-429 in 3 databases; (**C**) Pearson correlation analysis of miR-429 and nine overlapping genes; (**D**) Relative expression of DLC1 in human normal breast cell (MCF- 10A) and TNBC cell line (MDA-MB-468) was detected by Western Blot; (**E**) The binding site sequence of DLC1 3’UTR to miR-429 was predicted by TargetScan and validated by a dual luciferase assay; (**F**) qRT-PCR and Western blot (**G**) analysis of DLC1 expression in TNBC cell transfected with miR-429 mimic, mimic NC, miR-429 Inhibitor or Inhibitor NC. All figures are at a scale of 50μm. (* p < 0.05, ** p < 0.01, and *** p < 0.001).

Gene Expression Differential Analysis and target gene prediction were utilized to obtain the relevant genes concerning miR-429. Differential analysis of the mRNA expression profile of TCGA-TNBC revealed a total of 3948 significantly differentially expressed mRNAs (DEmRNAs), of which the upregulated and downregulated mRNAs numbered 2360 and 1588, respectively (Figure 3A). Afterward, the target gene prediction of miR-429 was performed by miRDB, Target Scan, and miRTarBase database, and finally, the significantly differentially down-regulated mRNAs were intersected with the predicted target genes of miR-429 to retrieve nine candidate genes (Figure 3B). Pearson correlation analysis of the nine candidate genes and miR-429 revealed a significant negative correlation of DLC1 with miR-429 (Figure 3C). In addition, cellular experiments depicted that protein expression of DLC1 was notably reduced in TNBC cells than in normal breast cells (Figure 3D).

The assumption of DLC1 acting as a direct target of miR-429 in TNBC was validated further through the TargetScan database. Herein, the miR-429 binding site on DLC1 was predicted and further verification was done through the dual luciferase assay (Figure 3E). The resulting data indicated that in contrast to the control, the dual luciferase activity ratio of miR-429 mimic and DLC1-3’UTR-WT co-transformation group was considerably diminished, whereas the ratio of miR-429 mimic and DLC1-3’UTR-MUT group did not vary notably. Therefore, miR-429 was found to bind to DLC1, as supported by the data. Moreover, western blot and qRT-PCR revealed that elevated miR-429 notably suppressed the protein and mRNA expression of DLC1, while its knockdown resulted in enhanced DLC1 expression (Figure 3F, 3G). The resulting data confirm that miR-429 targets and negatively regulates DLC1 expression in TNBC cells.

### miR-429 increases TNBC cell proliferation, migration, and invasion through degrading DLC1

**Figure 4:**
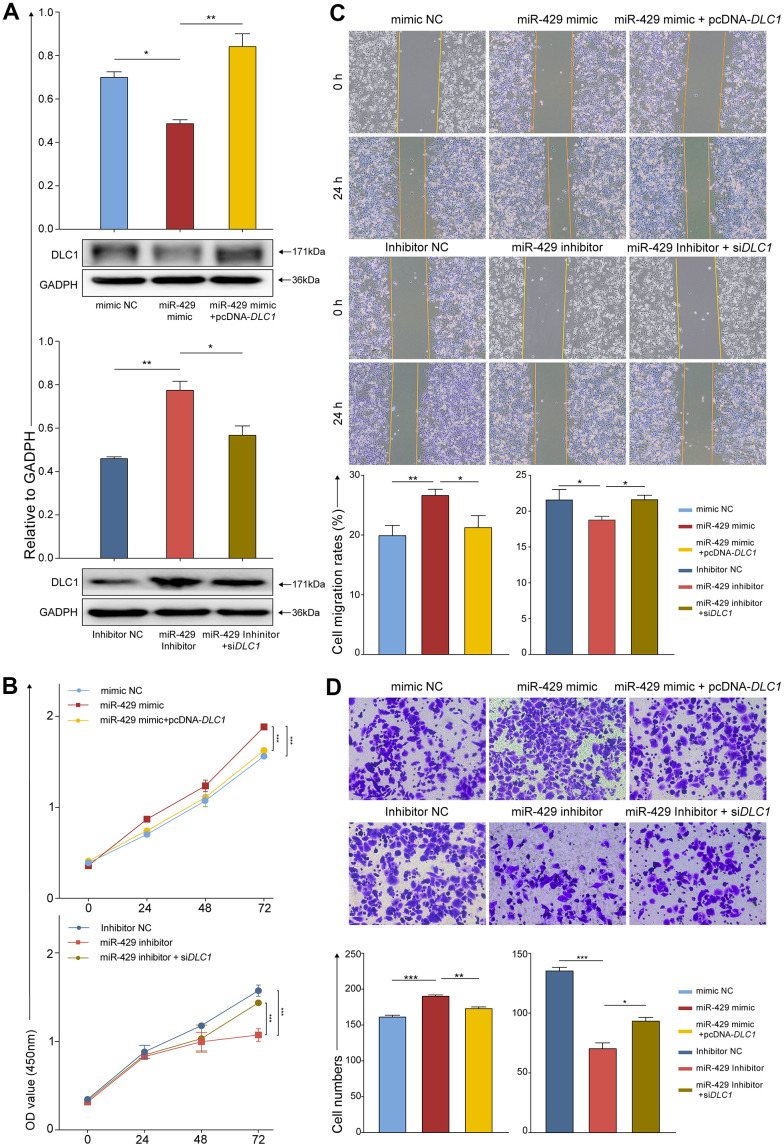
**miR-429 increases TNBC cell proliferation, migration, and invasion through degradation of DLC1.** (**A**) Protein levels of DLC1 relative to GADPH in cell assessed by Western Blot after transfection with indicated vectors, i.e., siRNA, miRNA, or inhibitor; (**B**) The cell proliferation was assessed by CCK-8 after transfection (0h, 24h, 48h,72h); (**C**) Wound healing assays were used to detect migration of cells after transfection (magnification, × 100). (**D**) The cell invasion was determined by transwell after transfection (magnification, × 200). All figures were 50 μm of scale bar. (* p < 0.05, ** p < 0.01, and *** p < 0.001).

Concerning additional confirmation of whether miR-429 exerts biological influence on TNBC cells by directly targeting DLC1, the pcDNA-*DLC1*, and the si *DLC1* were constructed. Afterward, MDA-MB-468 cells were divided into six groups for transfection: 1) mimic NC; 2) miR-429 mimic; 3) miR-429 mimic+pcDNA-*DLC1*; 4) inhibitor NC; 5) miR-429 inhibitor; and 6) miR-429 inhibitor+si *DLC1*. Subsequently, numerous follow-up experiments were performed. The resulting data exhibited that elevated miR-429 levels significantly decreased DLC1 protein expression in contrast to the control, whereas DLC1 protein expression was considerably increased in the concomitant transfection of the miR-429 mimic+pcDNA-*DLC1* group. In addition, DLC1 protein expression was notably elevated in the miR-429 inhibitor group in contrast to the inhibitor NC group, whereas DLC1 protein expression was reduced in the concomitant transfection of the miR-429 inhibitor+si *DLC1* group (Figure 4A).

As shown by the CCK-8 assay, the viability of the cell was notably enhanced in the miR-429 mimic group than in the mimic NC group and was partially reduced when miR-429 and DLC1 were simultaneously overexpressed. Similarly, in contrast to the inhibitor NC group, the viability of the cell was markedly lowered in the miR-429 inhibitor group, and was partially restored when miR-429 and DLC1 were simultaneously inhibited (Figure 4B). Wound healing and transwell assays indicated that the cell migrative and invasive of the miR-429 mimic group was notably increased in contrast to the mimic NC group, while this ability of the miR-429 mimic+pcDNA-*DLC1* group was reduced in contrast to the miR-429 mimic group. Considerably diminished migrative and invasive were depicted in the miR-429 inhibitor group than in the inhibitor NC group, and cell migration and invasion were partially restored when both miR-429 and DLC1 were inhibited (Figure 4C, 4D). The above experimental data suggest that miR-429 may negatively regulate DLC1 expression, which led to the development of TNBC.

### DLC1 functions as the tumor suppressor in TNBC

**Figure 5:**
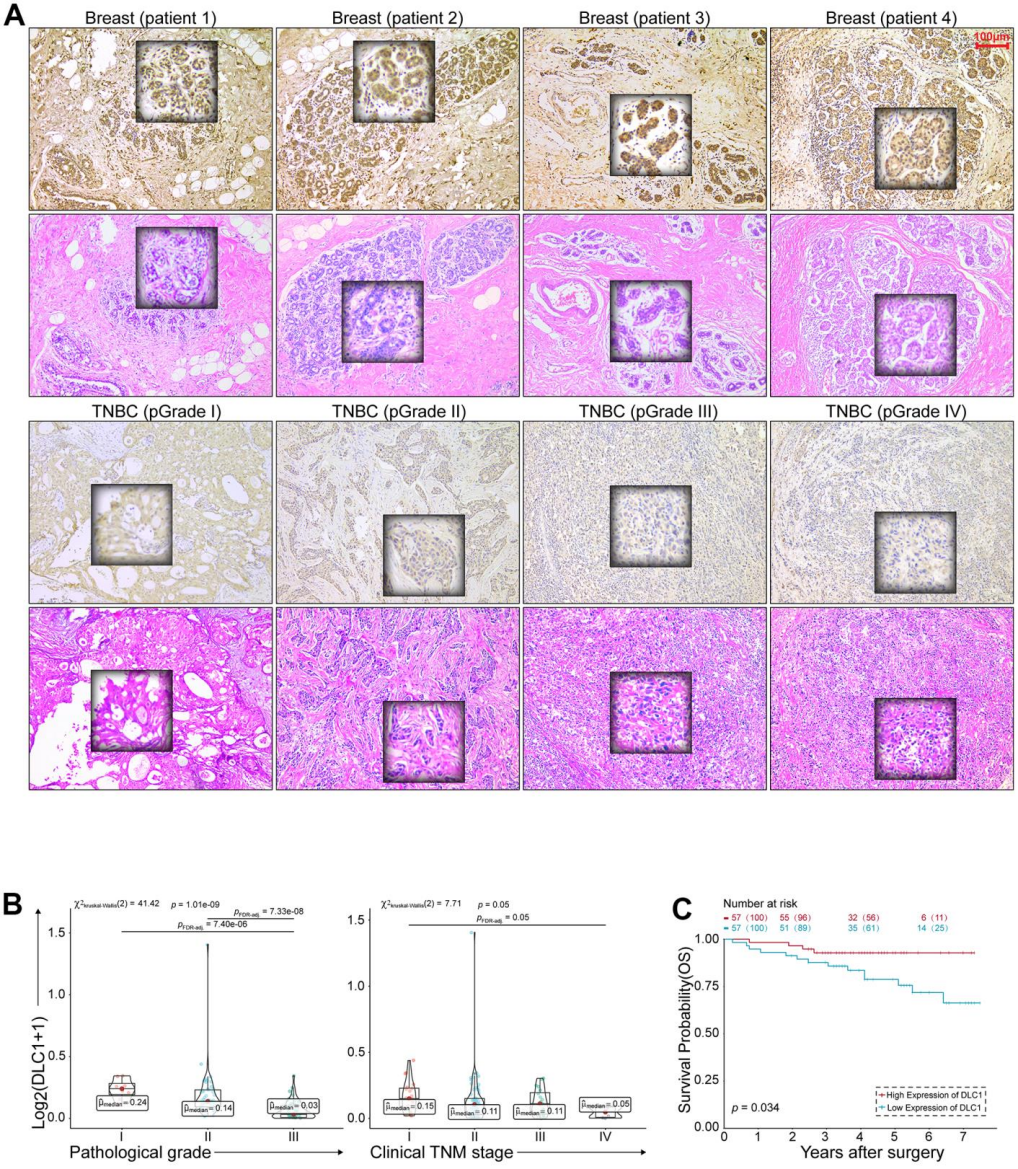
**DLC1 expression correlates with the anticancer properties of TNBC.** (**A**) HE and IHC staining of TNBC. Scale bar = 100 μm. (**B**) Correlation of DLC1 immunostaining intensity with clinical TNM stage and histopathological grade of TNBC, respectively. (**C**) Kaplan-Meier survival curves for overall survival of 114 patients with TNBC according to the DLC1 expression. Patients were stratified into high-expression and low-expression groups by median expression. (* p < 0.05, ** p < 0.01, and *** p < 0.001).

The clinical significance of DLC1 was evaluated by IHC in 114 TNBC archival tissues. The staining scores were evaluated by Image software and the clinical features were shown in Table 2. As shown in Figure 5A, the positive stain of DLC1 was in the cytoplasm of cells, A higher expression of DLC1 expression was shown in normal breast tissue compared to TNBC tissue. The staining intensity presented inverse correlations with clinical TNM stage (*p* = 0.05) and histopathological grade (p = 1.01e - 09) (Figure 5B). Kaplan-Meier survival curve indicated that the expression levels of DLC1 were positively correlated with the overall survival of patients with TNBC (Figure 5C). These results suggest that DLC1 may be an unfavourable factor for the occurrence and development of TNBC.

**Table 2:** Correlation between DLC1 expression and clinicopathological features in 114 TNBC patients.

**Characteristic**		**DLC1**		**P value**
		**Low**	**High**	
Age	<49.5	41	16	0.53
	≥49.5	39	18	
Ki-67(%)	≥20	64	31	0.07
	<20	12	7	
pGrade	I	6	2	1.01e-09
	II	35	19	
	III	29	23	
cTNM stage	I	12	8	0.05
	II	51	17	
	III	13	7	
	IV	3	3	

## DISCUSSION

Individuals with TNBC often develop drug resistance and have a high rate of distant recurrence and poor patient prognosis [17]. Therefore, it is imperative to explore the molecules associated with the development of TNBC and to examine new target molecules for the treatment of TNBC patients. There is growing evidence in recent years that miRNAs perform an important function in regulating the progression of various malignancies, and they have the potential to serve as both a novel diagnostic and a valuable therapeutic approach [18].

Previous reports have confirmed that miR-429 may perform in the capacity of an oncogene in a variety of tumors promoting tumor development. For instance, Machackova et al. [19] determined that elevated miR-429 levels existed in clear cell renal carcinoma tissues and negatively linked this elevated expression with the TNM stage. Individuals with increased expression had shortened disease-free survival and OS, suggesting the potential of miR-429 in the diagnosis and prognosis of individuals with renal cell carcinoma.

This research demonstrated the elevated expression of miR-429 in the cell lines and TNBC tissues and determined its notable influence on the prognosis of afflicted individuals. Hence, it is plausible to hypothesize that a possible association may exist between miR-429 and the malignant development of TNBC. To test this hypothesis, miR- 429 upregulation and downregulation models were established. The resulting data implies that highly expressed miR-429 may markedly enhance the migrative, invasive, and proliferative capability of TNBC cells, whereas downregulation of miR- 429 suppressed these processes.

Prior research has elaborated and assessed the molecular mechanisms of miR-429 in cancer. Guan et al. [20] identified a possible oncogenic mechanism of miR-429 in the development of ovarian malignancy, namely negatively regulating the inhibitor of growth family member 5 Gene (ING5). ING5 belongs to the oncogene ING family, whose downregulation is linked with ovarian carcinogenesis, metastasis, and angiogenesis [21]. miR-429 targets the 3’-UTR of ING5 directly and inhibits ING5 expression in ovarian cancer epithelial cells, thereby mediating tumorigenesis and cell proliferation. Han et al. [22] documented that miR-429 overexpression significantly enhanced the invasive and migratory capabilities of colorectal cancer cells, whereas suppression of miR-429 exhibited the opposite effect. The miR-429 can target and regulate the homologous cassette heterozygous gene 5 (HOXA5), which regulates the metastasis and proliferation of malignant colon cells which may be linked to the above phenomenon. Moreover, the activation of c-JUN and AVPR1A target genes were associated with the invasive expressions of breast cancer cells, including enhanced tumor proliferation and angiogenesis [23, 24]. Meanwhile, upregulation of ZFPM2 and ZFHX4 is associated with exacerbation and metastasis of BC [25, 26]. ZEB1 and ZEB2 mediate epithelial-to-mesenchymal transition (EMT) and metastatic progression in TNBC [27]. The inactivation of WASF3 and SESN1 was shown to play an essential role in the inhibition of invasion and metastasis in highly metastatic TNBC [28, 29]. These data suggest that by regulation of the expression of target genes, miR-429 may have a potential impact on promoting the growth of TNBC. As such, DLC1 was identified as a gene targeted by miR-429 through bioinformatic prediction.

DCL1 was initially identified as a gene missing or downregulated in primary hepatocellular carcinoma and exerts its carcinomatosis effects primarily utilizing the Rho-GTPase-activating protein (RhoGAP) structural domain [30–32]. Increasingly research has shown that DLC1 is linked with the development and progression of numerous malignancies such as ovarian, breast, and colorectal cancers, and is involved in suppressing metastasis [33, 34]. Zhang et al. [35] demonstrated the upregulation of miR-106b in colorectal cancer and its negative correlation with DLC1 expression. miR-106b, by targeting and regulating DLC1 3’UTR enhanced the migratory and invasive capabilities of the malignant colorectal cells. Concerning DLC1 expression, Ren et al. [36] documented its positive and negative correlation with OS and adverse prognosis, respectively, in individuals with breast cancer. This research implied that the expression of DLC1 was an unfavorable factor for the progression of TNBC. In addition, DLC1 showed a significant negative association and targeting relationship with miR-429. Through various experiments *in vitro*, it was shown that miR-429 negatively regulated DLC1 mRNA and protein expression through its binding to DLC1 3’UTR, which in turn promoted the migratory and invasive of TNBC cells as well as their proliferation. Furthermore, DLC1 could induce apoptosis, senescence and autophagy of cancer cells by regulating the EGFR/Akt/ NF-κB signalling pathway, and suppress the proliferation and migration of tumors by modulating DLC1/RhoA pathway [37]. Tripathi et al. [38] also reported that DLC1 suppressed NF-κB expression in prostate cancer due to its stabilization effect on adherens junctions. While NF-κB is highly implicated in TNBC chemoresistance, suggesting that miR-429 inhibition of DLC1 may break drug resistance.

In summary, this study implies that elevated levels of miR-429 can be found in TNBC, and its upregulation is a factor of poor prognosis in TNBC patients by TCGA. Herein, we confirmed that miR-429 promoted TNBC cell proliferation, migration, and invasion by targeted silencing DLC1, which helps to provide research support for the design of targeted drugs for TNBC. Nonetheless, it is important to note that this study has certain limitations, as the downstream signaling pathways linked to the inhibition of TNBC malignant development by DLC1 have not been thoroughly investigated. Our future research will focus on exploring these pathways in greater depth.
